# Comparative effectiveness of low-level laser therapy versus muscle energy technique among diabetic patients with frozen shoulder: a study protocol for a parallel group randomised controlled trial

**DOI:** 10.1186/s13018-024-04735-7

**Published:** 2024-04-30

**Authors:** Halima I. Hassan, Bashir Kaka, Fatima Bello, Francis Fatoye, Aminu A. Ibrahim

**Affiliations:** 1 Department of Physiotherapy, Faculty of Allied Health Sciences, College of Health Sciences, Bayero University, Kano, Kano State Nigeria; 2 Department of Physiotherapy, Ahmadu Bello University Teaching Hospital, Zaria, Kaduna State Nigeria; 3 Division of Physiotherapy, School of Health Sciences, College of Health Sciences, University of KwaZulu-Nata, Westville, Durban, South Africa; 4 Endocrinology Unit, Department of Internal Medicine, Ahmadu Bello University Teaching Hospital, Zaria, Kaduna State Nigeria; 5 Department of Health Professions, Faculty of Health, Psychology and Care, Manchester Metropolitan University, Manchester, UK; 6 Department of Physiotherapy, School of Basic Medical Sciences, Skyline University Nigeria, Kano, Kano State Nigeria

**Keywords:** Conventional therapeutic exercises, Diabetes mellitus, Frozen shoulder, Low-level laser therapy, Muscle energy technique

## Abstract

**Background:**

Diabetes mellitus is one of the fastest-growing health challenges of the twenty-first century with multifactorial impact including high rates of morbidity and mortality as well as increased healthcare costs. It is associated with musculoskeletal complications, with frozen shoulder being commonly reported. While low-level laser therapy (LLLT) and muscle energy technique (MET) are commonly used to manage  this condition, there remains a lack of agreement on the most effective approach, with limited research available on their comparative efficacy.

**Objectives:**

To evaluate the comparative effectiveness of LLLT versus MET among diabetic patients with frozen shoulder.

**Methods:**

This is a single-centre, prospective, single-blind, randomised controlled trial with three parallel groups to be conducted at Ahmadu Bello University Teaching Hospital, Zaria, Kaduna State, Nigeria. Sixty diabetic patients with frozen shoulder will be randomly assigned into LLLT group, MET group, or control group in a 1:1:1 ratio. All the groups will receive treatment three times weekly for 8 weeks. The primary outcome will be shoulder function and the secondary outcomes will include pain intensity, shoulder ROM, interleukin-6 (IL-6), depression, anxiety, and quality of life (QoL). All outcomes will be assessed at baseline, at post 8-week intervention, and at 3 months follow-up.

**Discussion:**

This will be the first randomised controlled trial to evaluate the comparative effectiveness of LLLT versus MET on both clinical and psychological parameters among diabetic patients with frozen shoulder. The findings of the study may provide evidence on the efficacy of these interventions and most likely, the optimal treatment approach for frozen shoulder related to diabetes, which may guide clinical practice.

*Trial Registration:* Pan African Clinical Trials Registry** (**PACTR202208562111554). Registered on August 10, 2022.

**Supplementary Information:**

The online version contains supplementary material available at 10.1186/s13018-024-04735-7.

## Background

Diabetes mellitus (DM) is one of the fastest-growing public health issues of the twenty-first century owing to its increasing prevalence rates, related morbidity and mortality, as well as health expenditures at global, regional and national levels [1, 2]. Approximately 537 million people are living with DM globally, and this number is projected to 643 million by 2030, and 783 million by 2045 [2]. Low-income countries, especially those in Sub-Saharan Africa are likely to bear one of the greatest burdens of DM epidemic as the number of individuals living with type 2 DM is expected to increase at the fastest rate in the coming decades. In 2021, South Africa was ranked the highest country for the number of people with DM (4.2 million) followed by Nigeria (3.6 million) [1]. The proportion of undiagnosed DM cases in Africa (67%) is almost twofold higher than that of developed countries (37%) [3], which may be linked to the high levels of morbidity and mortality at an early age in this continent [4].

Individuals with DM, especially those with poor glycemic control are at increased risk of debilitating and life-threatening complications resulting in increased disability, poor quality of life (QoL), reduced life expectancy, and considerable healthcare costs. Although microvascular and macrovascular complications are well-known and treated [5], musculoskeletal complications are also prevalent in DM [6–8]. Diabetics are 1.7–2.1 times more likely to experience musculoskeletal pain compared with non-diabetics [9], with a prevalence of 58.2% for musculoskeletal disorders (MSKD) [10]. Despite their impact on morbidity and pain [6], MSKD are generally overlooked compared to vascular complications, possibly because they are not life-threatening [11]. These complications stem from similar pathogenic factors as internal organ complications, including chronic low-grade inflammation [12].

A variety of MSKD have been well-documented to be associated with both type 1 and type 2 DM [7, 10]. The most commonly documented MSKD include carpel tunnel syndrome, Dupuytren's contracture, flexor tenosynovitis, frozen shoulder, limited joint mobility, and osteoarthritis [13, 14]. However, among the different disorders, fibrosing conditions affecting the hand were generally reported to be more prevalent (33.1%) followed by those affecting the shoulder (32%) [10].

Frozen shoulder is one of the common painful and disabling fibro-proliferative disorders characterized by a gradual onset of pain and limitation of glenohumeral range of motion (ROM) that can restrict activities of daily living without any radiological evidence [15, 16]. The odds of developing frozen shoulder for patients with DM was 3.7 times the odds for those without DM [17] and the overall prevalence of frozen shoulder among diabetics was estimated at around 13.4% (95% CI 10.2–17.2%) [18]. The impact of living with diabetic frozen shoulder is multifactorial including clinical factors such as severe pain, impaired mobility, and disability [19, 20] and psychosocial factors such as anxiety, depression, sleep deprivation, and altered domains of QoL [19, 21, 22]. Indeed, people with DM may experience worse outcomes from frozen shoulder than those without DM [23]. Frozen shoulder in diabetic patients is associated with the duration of DM and age [24], with those aged 40–59 years [25] and females [26] being more commonly affected.

The etiology of frozen shoulder in diabetes is not fully understood, but proposed mechanisms include impaired microcirculation and non-enzymatic glycosylation processes in the shoulder joint tissues [27]. Altered levels of inflammatory cytokines, such as interleukins, have also been linked to inflammation and fibrosis characteristic of frozen shoulder [28]. The management of frozen shoulder includes various approaches such as education, physiotherapy, medications, and surgery to alleviate pain and enhance ROM and function [29]. However, there is no consensus on the most effective single treatment approach, leading to a preference for combination therapies [30, 31].

Conventional therapeutic exercises (CTE) are typically prescribed alongside other interventions such as electrotherapy and mobilisation techniques to alleviate symptoms of frozen shoulder. Exercises regarded as CTE include Codman's or pendulum exercises, active and passive ROM, wall ladder exercises, capsular stretching, pulley, self-stretching, strengthening exercises, and shoulder mobilisation exercises [32, 33]. These exercises stimulate mechanoreceptors and improve joint lubrication and ROM thereby reducing inflammation and pain as well as enhancing function.

Among the several electrotherapy modalities employed in physiotherapy, low-level laser therapy (LLLT) emerges as a promising modality for the treatment of frozen shoulder owing to its analgesic, anti‐inflammatory and biostimulating effects [34]. A class IIIa LLLT with a power output of < 5mW or a class IIIb LLLT with a power output of < 500 mW is often applied to achieve optimal therapeutic effect [35]. However, LLLT is typically used in combination with other therapies such as exercise rather than in isolation. While moderate evidence indicates that LLLT offers added short-term pain relief benefits when combined with exercise, evidence for additional benefits on shoulder function and ROM is limited [36].

One hands-on technique commonly used by physiotherapists to manage MSKD such as frozen shoulder is the muscle energy technique (MET) [37, 38]. It is a class of gentle soft tissue manipulation applied to lengthen shortened, contracted, or spastic muscles, strengthen weak muscles, decrease edema and passive congestion, correct faulty joint positions, as well as mobilise restricted or hypomobile joints [39, 40]. As frozen shoulder often involves inflammation, reduced ROM and pain, the use of MET to address  these symptoms is crucial [41]. While MET has shown effectiveness in improving pain, disability, and ROM in various MSKDs, its efficacy in frozen shoulder specifically requires further evaluation [42]. Morever, existing trials on MET for frozen shoulder [43–46] have yielded conflicting results and lack methodological robustness.

Frozen shoulder constitutes a major burden in patients with diabetes, yet the most optimal treatment remains obscure. Although both LLLT and MET are used to manage frozen shoulder, the superiority of one intervention over the other has not been explored. Moreover, no study has evaluated the effect of either intervention on inflammatory biomarkers or psychological variables. Given that both LLLT and MET could alleviate pain and enhance function, which may result in improved psychological well-being, it is probable that such interventions could positively influence depression, anxiety as well as QoL. As there has been no investigation into the effectiveness of LLLT and MET and their comparison through a well-conducted randomised controlled trial (RCT) among diabetic patients with frozen shoulder, this study will be conducted to assess the comparative effectiveness of LLLT versus MET among diabetic patients with frozen shoulder.

## Objectives

The primary objective is to evaluate the effectiveness of an 8-week LLLT *versus* MET compared to CTE (control) on shoulder function among diabetic patients with frozen shoulder. The secondary objective is to evaluate the effectiveness of an 8-week LLLT *versus* MET compared to CTE on pain intensity, shoulder ROM, interleukin 6 (IL-6), depression, anxiety, and QoL.

### Hypotheses

We hypothesize that patients receiving either LLLT or MET would demonstrate superior improvement in terms of shoulder function, pain intensity, shoulder ROM, interleukin 6 (IL-6), depression, anxiety, and QoL compared to those receiving CTE.

## Materials and methods

### Trial design

**Fig. 1:**
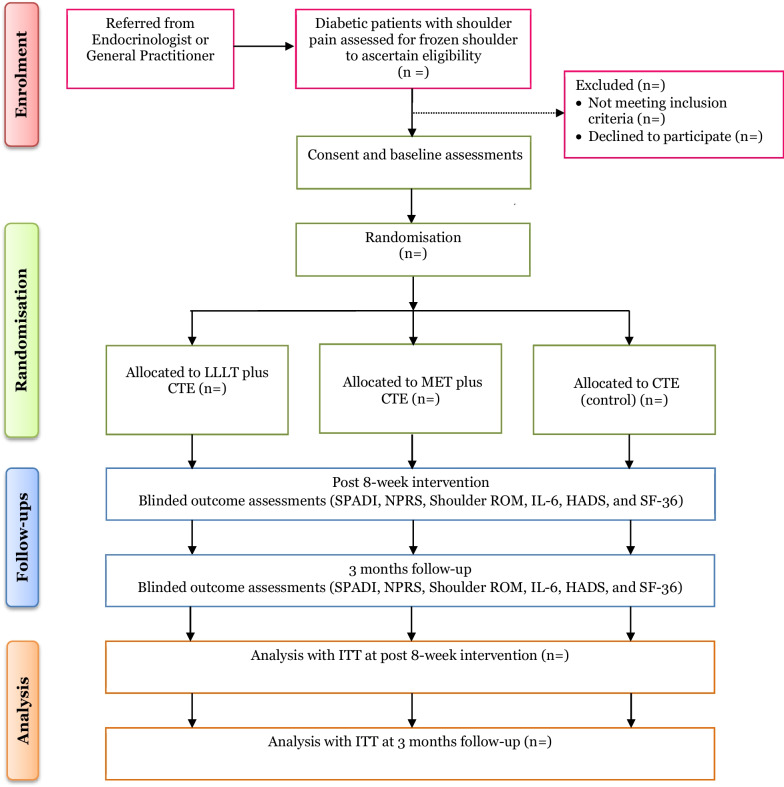
Flow of participants through the study

This study is a single-centre, prospective, single-blind, RCT with three parallel groups: LLLT, MET, and CTE (control) in a 1:1:1 ratio. This protocol was written in accordance with the Standard Protocol Items: Recommendations for Interventional Trials (SPIRIT) guidelines (Additional file 1). The flow of participants through the study is depicted in Fig. 1.

### Study settings

The study is conducted in the outpatient unit of the Physiotherapy Department at Ahmadu Bello University Teaching Hospital (ABUTH), Zaria, Kaduna State, Nigeria.

### Eligibility criteria

The following are the inclusion criteria for patient selection:

1. Both male and female diabetic patients (both type 1 and 2) aged 20 to 65 years old [8].
2. Patients with shoulder pain within the last 3 months (painful or first stage).
3. Patients with decreased active glenohumeral ROM of at least 20° or more in at least three movements: flexion < 144°, abduction < 120° and external rotation < 72° [47].
4. Patients having bilateral or unilateral shoulder symptoms.

The following are the exclusion criteria for patient selection:

1. Patients with a history of trauma to the shoulder.
2. Neurological involvement such as stroke, brachial plexus injury, Parkinson's disease, and cervical spine injury with or without radiculopathy.
3. History of surgery to the shoulder, malignancy, or tumour of the shoulder.
4. Shoulder arthritis, rotator cuff tear, or other shoulder ligamentous injury.

### Informed consent

The physiotherapist responsible for eligibility assessment will obtain written informed consent from the participants after explaining the study procedure. During the study period, all potential participants will have the opportunity to withdraw at any time point during the study period.

### Additional consent provisions for collection and use of participant data and biological specimens

Not applicable. Data and biological specimens will not be collected for ancillary studies.

### Explanation for the choice of comparators

Therapeutic exercises including flexibility and strength training are commonly used by physiotherapists to manage frozen shoulder and, hence considered to be an established treatment or standard of care. Our trial is designed to compare two experimental interventions (LLLT and MET) with therapeutic exercises– here referred to as CTE as the control intervention. The active control group will help to mitigate the possibility that improvements seen in the experimental groups are due to a placebo effect.

### Intervention description

The control group will receive CTE only. In the LLLT group, participants will receive LLLT followed by CTE. Similarly, participants allocated to MET group will receive MET followed by the same CTE. All interventions will be administered three times per week for 8 weeks, equivalent to a total of 24 treatment sessions. The participants will be treated on alternate days to minimise group contamination. The primary researcher who has over 13 years of clinical experience in musculoskeletal physiotherapy will administer all the interventions. Another licensed physiotherapist with 5 years of clinical experience will assist in administering the CTE. For patients presenting with bilateral affectation, both shoulders will be treated.

### Conventional therapeutic exercises (CTE)

Eight CTE aiming to improve shoulder ROM (i.e. flexion, abduction adduction, and external and internal rotation) and strength will be administered. Each exercise will be repeated 5–10 times per session. Progression of exercise will be based on the patient's pain thresholds or tolerance. The exercises include:

1. Pendulum exercise: The participant’s position will be standing or prone lying. The patient leans forward in standing by supporting the unaffected shoulder on a plinth. The affected shoulder is hung down parallel with the trunk and then swings forward and backward side to side or around in circles.
2. Arm overhead exercise: The participant lies supine and supports the affected arm with the unaffected hand. The participant then lifts the affected arm overhead.
3. Finger wall ladder: The participant slowly walks his finger up the wall and slowly lowers the arm.
4. Twisting arm outwards: The participant lies supine with the knees bent, and then places the hand behind the neck or head and lets elbows fall outwards.
5. Overhead pulley: The participant sits on a chair, and grasps one handle with the affected side overhead with the palm facing inward. The unaffected hand grasps the other handle with the elbow flexed, and gently stretches the affected shoulder overhead by extending the elbow.
6. Cross-body reach exercises: The participant stands and takes the affected hand across towards the opposite shoulder, and then stretches gently by pulling the affected arm at the elbow and holds for 10–15 s.
7. Hand behind-back exercise: The participant stands with arms by side, grasps the affected wrist, stretches the hand towards the unaffected shoulder, and slides the arm up the back. The exercise can be progressed to the use of a towel.
8. Outward rotation exercise: The participant stands, holds a rubber band or TheraBand with the elbow at 90°, and then rotates the affected arm outward while holding for 5 s.

### Low-level laser therapy (LLLT)

Laser treatment will be applied using a Class 3B Laser therapy unit (I-TECH LA 500), with a wavelength of 810 nm, continuous output power of 60 Mw, and spot size/area of 0.5 cm^2^, similar to the protocol described by Stergioulas [48]. Painful areas of the shoulder such as the subacromial space, bicep anchor, axillary pouch, anterior shoulder capsule, and posterior shoulder capsule will be irradiated with a power density of 5.4 j/cm^2^ and duty cycle of 50%. The application time will be 30 s per area. Prior to application, the area to be treated will be cleaned with methylated spirit to minimize reflection from skin and probe [48]. Rotation interval and probe will be perpendicular to the circumscribed area thereby preventing energy loss due to divergence. Both the therapist and participant will wear protective eye goggles during the treatment as a safety measure.

### Muscle energy technique (MET)

This will be administered for restriction in shoulder flexion, abduction, and external rotation. The treatment protocol procedure described in this study is identical to that described in the literature [36].

- MET for shoulder flexion restriction: The participant will be in a side-lying position with the affected shoulder uppermost and the therapist will stand at chest level of the participant with one hand holding the patient's forearm and the other hand stabilising the participant's clavicle and scapula. While maintaining the participant's uppermost shoulder into flexion passively, the patient will be instructed to pull the elbow towards the feet by utilising approximately 20% maximal effort. The therapist will firmly resist this effort for 7–10 s and then move the shoulder arm into further flexion to the next restriction barrier. The treatment protocol will be repeated 3 times per session. The targeted muscles are the deltoid, pectoralis major, biceps brachii, subscapularis, and latissimus dorsi.
- MET for shoulder abduction restriction: The participant will be in a supine and the therapist will stand at the side with one hand holding the participant's flexed elbow and the other hand stabilising the patient's clavicle and scapula. The therapist will then horizontally adduct the participant's shoulder to the initial point of pain or barrier and instruct the participants to pull the arm while utilising approximately 20% maximal effort. The therapist will firmly resist this effort for 7–10 s and instruct the participant to relax, and while exhaling, the therapist, using his contact on the elbow, moves the participant’s shoulder further into abduction. The treatment protocol will be repeated 3 times per session. The targeted muscles are the deltoid, supraspinatus, infraspinatus, teres minor, and subscapularis.
- MET for shoulder external rotation restriction: The participant will be in a supine position with the arm abducted to 90°, the elbow flexed to 90°, the forearm in internal rotation, and the palm facing upward. If the participant cannot abduct or flex to 90°, the available range will be the starting point and gradually increase as the participant improves. The therapist stands at the side with one hand holding the participant's forearm and the other hand stabilising the participant's clavicle and scapula. While the participant's entire arm is resting at the restriction barrier, with gravity as its counterweight, the participant will be instructed to raise the forearm slightly, against minimal resistance provided by the therapist, for 7–10 s. Following relaxation, gravity or slight assistance from the therapist takes the arm into greater external rotation, through the barrier, where it will be held for 30 s. The treatment protocol will be repeated 3 times per session. The targeted muscles are the infraspinatus, teres minor, and posterior deltoid.

### Criteria for discontinuing or modifying allocated interventions

No provisions for changing the trial arm allocation. However, the criteria for discontinuing the allocated interventions will be participant withdrawal of consent at any point of the study without providing reasons and participant experiencing worsening of condition related to other diabetic complications. Any data collected up to the point of withdrawal will be included in the study and intention-to-treat analyses will be conducted.

### Strategies to improve adherence to interventions

To ensure intervention adherence during the trial period, all participants will be well informed during the consent process about the importance of completing all treatment sessions and outcome measurements post 8-week intervention and at 3 months follow-up.

### Relevant concomitant care permitted or prohibited during trial

Relevant concomitant care and interventions will be permissible during the trial other than administration of other experimental treatments that might influence or bias the study outcomes.

### Provisions for post-trial care

We will document any adverse events (AEs) within the current study and ensure that appropriate care is provided to study participants if necessary.

### Outcomes

Two independent assessors (licensed physiotherapists) who will be trained by the primary researcher (HIH) before participating in the trial and blinded to group allocation will assess all outcome measures prior to randomisation. For patients with bilateral shoulder affectation, the more painful side will be chosen for outcome assessment.

### Baseline data collection

Sociodemographic characteristics (e.g. age, gender, marital status, education level, occupational level, height, weight, and body mass index) and clinical characteristics (e.g. duration of DM, type of DM, affected side, smoking status, alcohol status, presence of comorbidity, fasting glucose level and two-hour post-prandial blood glucose) will be obtained and recorded using a prepared study proforma. All participants will be given a specific ID for recognition.

### Primary outcome measure

The primary outcome measure is the shoulder function to be assessed using the Shoulder Pain and Disability Index (SPADI). It consists of 13 items divided into two subscales (5 items for shoulder pain and 8 items for shoulder disability). Each item is scored on a 0–10 numerical rating scale, where 0 denotes the best score and 10 denotes the worst score [49]. The scores of each subscale are added up and converted into a score out of 100, with higher scores indicating greater pain and disability [50]. The SPADI is a valid, reliable, and responsive measure of shoulder pain and disability [50–52]. Both the English [53] and Hausa (unpublished data) versions will be used in this study.

### Secondary outcome measures

The secondary outcome measures include pain intensity, shoulder ROM, depression, anxiety, QoL, and IL-6.

1. Pain intensity: The participants’ shoulder pain intensity will be assessed using the Numerical Pain Rating Scale (NPRS). It is an 11-point Likert scale, with 0 representing “no pain” and 10 representing “worst imaginable pain". The participants will be asked to mark or cycle the best point that represents the greatest pain they experienced at the time of assessment. The NPRS is a valid, reliable, and responsive measure of pain intensity in patients with shoulder pain [54, 55]. Both the English [56] and Hausa [57] versions will be used in this study.
2. Shoulder ROM: The participants’ painful shoulder range of flexion, abduction, and external rotation will be measured in a supine position using a 12-inch, 360-degree goniometer (G30, China) in line with standardised instructions [58, 59]. The universal goniometer was reported to have adequate reliability with intraclass correlation coefficients (ICC) of 0.91 to 0.99) in measuring shoulder ROM [60]. In the present study, the reliability of the shoulder ROM measurements will be checked by an external assessor.
3. Interleukin-6: Venous blood samples of the participants will be obtained and processed at the Immunology Unit of Medical laboratory Department, ABUTH, Zaria. The participant's venous blood (from the median cubital vein or the cephalic vein) using plain bottles containing 50 µL of aprotinin will be collected by a well-trained laboratory technician and the plasma will be stored at − 80 °C until analysis. The serum level of IL-6 will be then processed by a medical laboratory scientist, using enzyme-linked immunosorbent assay (ELISA) method with high-sensitivity kits (Quantikine®HS, R&D Systems Minneapolis, U.S.).
4. Anxiety and depression: Participants' levels of depression and anxiety will be assessed using the Hospital Anxiety and Depression Scale (HADS). It consists of 14 items divided into 2 subscales, with 7 items for anxiety and 7 items for depression. Each item is rated on a 4-point Likert scale (range 0–3). The total score is the sum of the 14 items, and for each subscale, the score is the sum of the respective 7 items ranging from 0–21. Scores of 0–7 indicate 'no case', 8–10 indicate 'possible case', and > 11 indicate a 'probable case of anxiety/depression' [61]. The HADS is a valid, reliable, and responsive measure of anxiety and depression in different patient populations as well as the general population [62]. Both the English [61] and Hausa (unpublished data) versions will be used in this study.
5. Quality of life (QoL): Participants’ QoL will be assessed using the 36-item short-form health survey (SF-36). The questionnaire consists of 36 items that are clustered to yield 8 domains of health status: physical functioning (PF), role-physical (RP), bodily pain (BP), general health (GH), vitality (VT), social functioning (SF), role-emotional (RE), and mental health (MH). Two summary measures, namely the physical component summary (PCS-12) and mental component summary (MCS-12) scores can be obtained from the 8 domains. Each domain and summary scale is scored 0–100, with higher scores indicating higher levels of function and/or better health [63, 64]. The scoring of the SF-36 will be computed using standard published guidelines [65]. The SF-36 has shown good psychometric properties in patients with shoulder disorders [66]. Both the English [63] and Hausa (unpublished data) versions will be used in this study.

### Participant timeline

The participant timeline is based on the SPIRIT statement and is provided in Table 1.

**Table 1:**
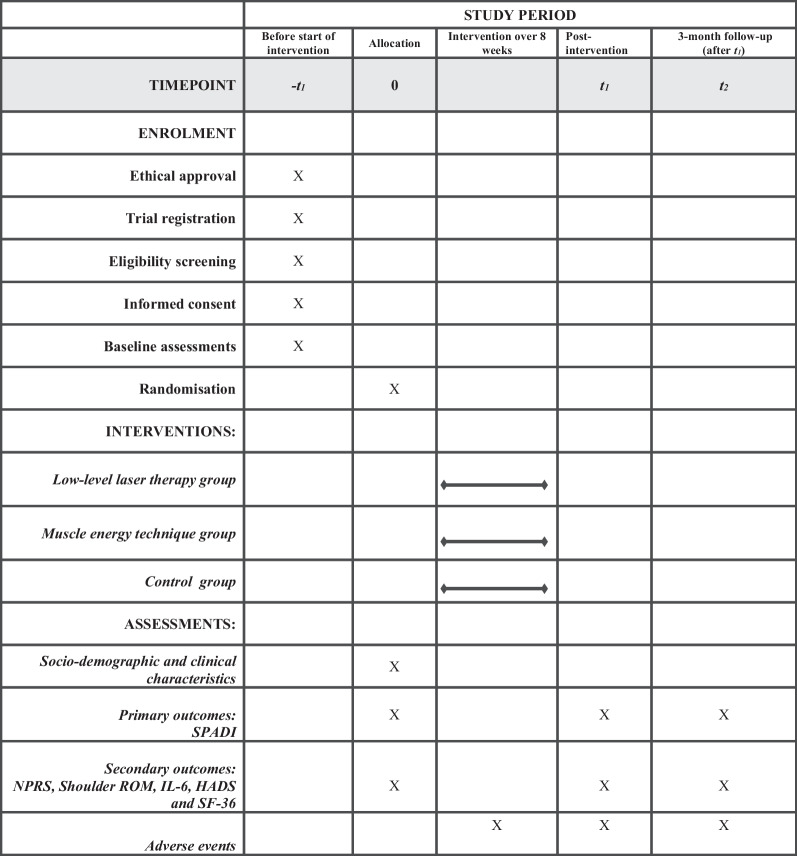
SPIRIT figure: time points for enrolment, interventions, and assessment

SPADI, Shoulder Pain and Disability Index; NPRS, Numerical Pain Rating Scale; ROM, Range of motion; IL-6, Interleukin 6; HADS, Hospital Depression and Anxiety Scale; SF-36, 36-item short-form health survey

### Sample size estimation

To our knowledge, no trial could be found evaluating the comparative effects of LLLT and MET among diabetic patients with frozen shoulder. However, given that the minimal clinically important difference (MCID) of the SPADI has been reported to be 8.0% points [67], the required sample size was estimated to detect a minimum difference of 8.0% (standard deviation [SD] = 10.0%) points in SPADI between the experimental groups (LLLT and MET) and control group (CTE) means post-intervention. Using the following parameters: F-test, repeated measures, between-subjects analysis of variance (ANOVA); alpha (*α*) level of 0.05; power of 0.80; effect size of 0.38 when accounting for 3 repeated measures; and correlation among repeated measures of 0.5, a total of 48 participants (16 participants per group) will be required for the trial. While anticipating an attrition rate of 25% (*n* = 12), the final sample size is 60, with 20 participants per group. Calculations were carried out using G Power 3.1.9.2 software [68].

### Recruitment

Diabetic patients with shoulder pain at ABUTH, Zaria will be recruited from the endocrine clinic or general outpatient department upon referral by a consultant endocrinologist or general practitioner for physiotherapy. Recruitment will be through adverts posted on clinic notice boards. Eligibility will be determined by an experienced musculoskeletal physiotherapist using standardised clinical diagnosis, including inspection, palpation, and examination of ROM and provocative maneuvers. Written informed consent will be obtained from eligible participants by the physiotherapist after explaining the study procedure.

### Randomisation: sequence generation, allocation concealment and blinding

Eligible participants who provide consent for participation will be randomly assigned to LLLT group, MET group, or control group with an allocation ratio of 1:1:1 using block randomisation procedure. To achieve balance in the allocation of participants to the three treatment arms, a block randomisation sequence with variable block sizes will be generated using computed-generated random numbers. No stratification will be used. The allocation sequence will be concealed using consecutive numbered, sealed, and opaque envelopes. The block sizes and randomisation list will be concealed until the end of the study. An independent physiotherapist at the Department of Physiotherapy, ABUTH, Zaria, who will not be involved in any other aspects of the study will be responsible for the allocation of participants to the various study arms. Physiotherapists undertaking outcome assessments and statistician performing data analyses will be blinded to group allocation.

### Procedure for unblinding if needed

Due to the nature of the exercise intervention in this study, neither participants nor treatment providers will be blinded. However, participants will be informed about their group allocation after the study is completed.

### Plans for assessment and collection of outcomes

All outcome measures will be collected at inclusion (baseline), at post 8-week intervention, and 3 months follow-up (Table 1).

### Plans to promote participant retention and complete follow-up

To minimise loss from follow-up, participants' contact information will be validated at each time point. They will be reminded of their follow-ups through phone calls at regular intervals.

### Data management

Participants' data will be stored in a logbook and electronically using Microsoft Excel sheets on a computer hard drive. The data will be checked for errors before entry.

### Confidentiality

All participants will be recognised only by their ID codes and their information will be kept strictly confidential, and only accessed by members of the trial team or ethics committee. Open request, only anonymised data will be made available to other researchers to enable international prospective analyses.

### Plans for collection, laboratory evaluation, and storage of biological specimens for genetic or molecular analysis in this trial/future use

This is not applicable as no biological specimens will be collected.

### Statistical analysis

All statistical procedures will be conducted using SPSS version 24.0 (IBM Co., Armonk, NY, USA) at a probability level of 0.05. Data distribution will be examined statistically using the Kolmogorov–Smirnov and Shapiro–Wilk test, and graphically using histograms, Q-Q plots, and Boxplots. The Levene test will be applied to check for homogeneity of variance. In case of skewed distribution, data transformations using log or square root transformation will be used. Continuous variables will be presented by mean and SD whereas categorical variables will be presented using frequency and percentage. Baseline comparison of continuous variables among the groups will be performed using One-way ANOVA and for categorical variables using the chi-square (χ^2^) test.

### Statistical methods for primary and secondary outcomes

The intention-to-treat principle will be the main analysis and will be conducted by including each participant's available data according to original allocation and irrespective of the level of attendance. A mixed between–within subjects ANOVA will be used to evaluate time effect (baseline, 8 weeks, 3 months), group effect (LLLT group, MET group, control group), and group-by-time interaction effect for all outcome measures. Post hoc analysis using Bonferonni correction will be applied for pairwise comparisons for significant differences detected in the primary outcome measures. Fisher's least significant difference (equivalent to no adjustment) will be applied to the secondary outcome measures. Effect size will be computed to describe the magnitude of change in all the outcomes. Potential confounding variables such as age, gender, BMI, and duration of DM will be controlled in the main analyses.

### Methods for additional analyses (e.g. subgroup analyses)

We plan to conduct responder analyses for the proportion of participants attaining clinically relevant improvement (≥ 8.0% points improvement from baseline) in shoulder function (SPADI scores) [67] post-intervention and at 3 months follow-up. Participants will be dichotomised into two “improved” (≥ 8.0% points) and “not improved” (< 8.0% points). A χ^2^ test will be used for comparison of the proportion of participants attaining or not attaining clinically relevant improvement in each study arm.

### Methods in analysis to handle protocol non-adherence and any statistical methods to handle missing data

As sensitivity analyses, per-protocol analyses will be conducted using data from participants with fully available data and no protocol violations. The per-protocol dataset will be predefined in the statistical analysis plan of the study. Multiple imputations by chained equations [69] will be used to handle missing data.

### Plans to give access to the full protocol, participant-level data, and statistical code

The protocol, participant-level data, and statistical code that support the findings of this study will be made available from the corresponding author, upon reasonable request.

### Composition of the coordinating center and trial steering committee

The principal investigator (HIH) and two other authors (BK and FB) constitute the trial steering committee (TSC) and will be responsible for monitoring patients’ recruitment, treatment, attrition, and progress of the trial monthly.

### Composition of the data monitoring committee, its role, and reporting structure

This trial has no external data monitoring committee since it involves low-risk interventions.

### Adverse event reporting and harms

Both CTE and MET are well-established interventions in medical rehabilitation, implying a low likelihood of serious adverse events (AEs) to occur with these interventions. However, non-serious AEs such as muscle soreness, fatigue, dizziness, or exacerbated muscle or joint pain may occur. LLLT is also considered a safe intervention when used appropriately, with no documented serious AEs. Participants will be informed of the potential for both serious and non-serious AEs before treatment. Any AEs will be reported to the primary researcher for assessment, potential participant withdrawal, and further action. Additionally, reports will be submitted to the Health Research Ethics Committee of ABUTH, Zaria, Kaduna State, Nigeria, and reviewed by a co-investigator.

### Frequency and plans for auditing trial conduct

The TSC will meet monthly to discuss issues with recruitment, protocol adherence, and follow-up of participants.

### Plans for communicating important protocol amendments to relevant parties (e.g., trial participants, ethical committees)

If there are any justifiable protocol modifications, the TSC will meet to discuss the importance of the proposed changes. The ethical committee will be communicated and updated in the Pan African Clinical Trials Registry. Amendments will be undertaken only after approval of all committees.

### Dissemination plans

The results of this trial will be disseminated to the participants, health care professionals, the public, patient advocacy groups, and other relevant groups through publications in peer-reviewed journals, conferences, press releases, and public talks regardless of whether the results are positive, negative, or inconclusive. The patient advocacy group will help in giving the relevant information to patients and their families concerning frozen shoulder in diabetes and the most effective treatment.

## Discussion

Frozen shoulder is a common and burdensome musculoskeletal complication among diabetic patients but it seems to be generally less prioritised and poorly treated [11]. This oversight may be due to challenges in managing diabetes-related complications, prioritising glycemic control, and healthcare providers' limited awareness of musculoskeletal issues in diabetic patients.

Despite the availability of various physiotherapy interventions for frozen shoulder, there remains a notable lack of substantial evidence supporting the superiority of one intervention over another [29]. While both LLLT and MET are commonly employed in the management of frozen shoulder, the comparative effectiveness of these interventions remains unexplored. Additionally, there is a notable gap in the literature regarding the evaluation of the effectiveness of either intervention on inflammatory biomarkers and psychological variables. As such, a comprehensive investigation into the relative efficacy of LLLT and MET for frozen shoulder treatment, encompassing their effects on both clinical outcomes and potential underlying mechanisms, is warranted. This would not only provide insight into the most  optimal treatment approach for improving function in frozen shoulder but also illuminate the impact of these interventions on inflammatory cytokines  and psychological outcomes.

We hypothesize that patients undergoing 8-week LLLT or MET interventions will exhibit superior improvements in all outcomes compared to those undergoing 8-week CTE post-intervention and at 3 months follow-up. By elucidating the most optimal treatment approach for frozen shoulder in diabetic patients, our study could guide clinical practice.

### Trial status

The study opened to participant recruitment on 2 February 2023. It is anticipated that recruitment will end by May 2024 and data collection (final follow-up) will be completed in August 2024.

### Trial registration

This trial was registered at the Pan African Clinical Trials Registry (https://pactr.samrc.ac.za/) on August 10, 2022 (registration number: PACTR202208562111554).

### Supplementary Information

**Additional file 1.** SPIRIT 2013 Checklist: Recommended items to address in a clinical trial protocol and related documents*.

## Data Availability

The authors will have access to the datasets. There is no plan to provide public access to the data used for this trial. However, data will be available from the authors upon reasonable request.

## Abbreviations

ABUTH: Ahmadu Bello University Teaching Hospital
ANOVA: Analysis of variance
AE: Adverse event
CI: Confidence interval
CTE: Conventional therapeutic exercises
DM: Diabetes mellitus
HADS: Hospital Anxiety and Depression Scale
IL-6: Interleukin 6
MET: Muscle energy technique
MSKD: Musculoskeletal disorders
LLLT: Low-level laser therapy
RCT: Randomised controlled trial
QoL: Quality of life
SF-36: 36-Item short form health survey
SPADI: Shoulder Pain and Disability Index
